# Trimodality bladder-sparing approach without neoadjuvant chemotherapy for node-negative localized muscle-invasive urinary bladder cancer resulted in comparable cystectomy-free survival

**DOI:** 10.1186/1748-717X-9-213

**Published:** 2014-09-24

**Authors:** Cheng-Yen Lee, Kai-Lin Yang, Hui-Ling Ko, Rong-Yau Huang, Pei-Pin Tsai, Ming-Tsun Chen, Yi-Chia Lin, Thomas I-Sheng Hwang, Guang-Dar Juang, Kwan-Hwa Chi

**Affiliations:** Department of Radiation Therapy and Oncology, Shin Kong Wu Ho-Su Memorial Hospital, No. 95, Wen-Chang Road, Shih-Lin District, Taipei City, Taiwan; Division of Urology, Department of Surgery, Shin Kong Wu Ho-Su Memorial Hospital, No. 95, Wen-Chang Road, Shih-Lin District, Taipei City, Taiwan; Shu-Tien Urology Ophthalmology Clinic, No. 276, Sec. 2, Jianguo South Road, Taipei City, Taiwan; School of Medicine, Fu Jen Catholic University, No. 510, Chung-Cheng Road, Hsin-Chuang, New Taipei City, Taiwan; School of Medicine and Institute of Biomedical Imaging and Radiological Sciences, National Yang-Ming University, No. 155, Sec. 2, Linong Street, Beitou District, Taipei City, Taiwan

**Keywords:** Urinary bladder cancer, Chemoradiation, Trimodality, Organ preservation

## Abstract

**Background:**

To retrospectively review the efficacy and organ preservation experience for muscle-invasive bladder cancer by trimodality therapy at our institution.

**Methods:**

Between July 2004 and February 2012, seventy patients (M/F = 55/15; median age = 69 years) of lymph node negative localized muscle-invasive bladder cancer were treated primarily with trimodality approach including transurethral resection of bladder tumor (TURBT) prior to combined chemotherapy and radiotherapy (CCRT). Radiotherapy consisted of initial large field size irradiation with 3D conformal technique (3D-CRT), followed by cone-down tumor bed boost with intensity modulated radiotherapy (IMRT) technique. The median total doses delivered to bladder tumor bed and whole bladder were 59.4Gy and 40.0Gy, respectively. No patient received neoadjuvant chemotherapy (NAC). Weekly cisplatin was administered during radiotherapy. Toxicity was scored according to the RTOG criteria. Tumor response was evaluated both cystoscopically and radiographically 3 months after treatment.

**Results:**

The numbers of patients with T2, T3 and T4 lesions were 41, 16 and 13, respectively. Overall survival (OS) and progression-free survival (PFS) at 2 and 5 year were 65.7%, 51.9% and 50.8%, 39.9%, respectively, after a median follow-up time of 24 months. Local-regional control and distant metastasis free survival at 2 year were 69.8% and 73.5%, respectively. Complete response (CR) rate assessed three month after CCRT was 78.1%. Ten patients (20%) had local recurrence after initial CR (n = 50), 3 of them were superficial recurrence. One patient underwent radical cystectomy after recurrence. The overall 5-year bladder intact survival was 49.0% (95% CI, 35.5% to 62.5%). Acute toxicities were limited to grade 1-2. One patient developed late grade 3 GU toxicity.

**Conclusions:**

Our result suggested that trimodality bladder-sparing approach without NAC or dose-intensification could be well-tolerated with a high CR rate and bladder preserving rate for muscle-invasive bladder cancer.

## Background

Historically, surgery was the main treatment of muscle-invasive bladder cancer. Radical cystectomy with urinary diversion and pelvic lymph node dissection resulted in 5-year pelvic control rate of 80–90% and 5-year OS of 40–60% [1]. Radical cystectomy and partial cystectomy (in selected cases) are frequently regarded as standard of care in contemporary management of muscle-invasive bladder cancer, where bladder-sparing radiotherapy was used as an alternative. Compared with radiotherapy alone, an OS benefit was observed with radical surgery in patients with muscle-invasive bladder cancer [2]. The introduction of bladder sparing therapy with or without NAC besides CCRT had been pioneered by Tester et al [3], Shipley et al [4] and Rodel et al [5]. CCRT has soon be regarded as the mainstream of organ preservation therapy instead of RT alone [6]. Although intriguing, the superiority of NAC has not been proven in randomized trial [4]. However, most trimodality treatment consisted of NAC followed by CCRT, with radical cystectomy reserved for incomplete responders in these reports [7]. Overall, the 5-year OS was approximately 50% with three-quarters of them maintaining functional bladders [7]. A trend toward increasing the intensity of chemotherapy during CCRT, such as the addition of vinblastine, paclitaxel and gemcitabine to cisplatin [8–11], and escalation of biological radiation dosage via hyperfractionation and hypofractionation was noted [9, 12]. However, late pelvic toxicity are concerning. In spite of these encouraging results and possible positive impact on quality of life [13], widespread use of this technique was still limited. In a report by Fedeli et al, the majority of muscle invasive bladder cancer in the United States were still treated with cystectomy (42.9%), while the minority were treated radiation therapy (16.6%) [14].

We hereby report our institutional experience on bladder preservation using upfront CCRT without NAC or adjuvant chemotherapy, with IMRT boost after whole bladder irradiation by 3D-CRT.

## Methods

### Patient characteristics

Between July 2004 and February 2012, 91 patients diagnosed with muscle-invasive (T2 to T4) bladder cancer who were treated with curative intent in our institution were retrospectively reviewed. Patients with pelvic lymph node metastases and distant metastases, as detected by computed tomography (CT), magnetic resonance imaging (MRI) or other modalities before RT were excluded in this review. Seventy patients with newly diagnosed or recurrent disease after initial TURBT treated with CCRT after TURBT or partial cystectomy were included in this analysis. This review was approved by the institutional review board of our hospital (No.20130506R). T-category was re-assigned according to the American Joint Committee on Cancer (AJCC) tumor-node-metastasis classification of 2010. The majority of patients included in the study had pathological evidence of tumoral invasion of the muscular layer, either after a TURBT (n = 64; 91.4%) or partial cystectomy (n = 4, 5.7%). The remaining patients (n = 2; 2.9%) received only biopsy, but had radiographical evidence of T4 disease. The majority of the population had urothelial cell carcinoma (n = 65, 92.9%). 55 of the patients (78.6%) had high grade disease on pathology.

### Treatment

Patients were referred from urologist. Those patients were not candidates for radical cystectomy because of comorbidities or personal preference. Radiotherapy was performed 4 to 8 weeks after TURBT or partial cystectomy. Planning CT scan with 3-mm slices was acquired for all patients undergoing RT. The patients were simulated in supine position with distended bladder. They were instructed to void and take a fixed amount of fluid (350-500 mL) thirty minutes prior to both simulation and treatment. Large field irradiation to subclinical disease by 3D-CRT followed by tumor-directed boost by IMRT was our treatment guideline. High risk clinical target volumes (CTV-H) were defined as preoperative tumor bed, which were delineated on the planning CT with respect to preoperative MRI or CT images registered by image fusion. In those who underwent partial cystectomy, the positions of surgical clips needed to be taken into consideration for delineating CTV-H. In the presence of gross residual tumor, the gross tumor volume (GTV) was delineated on planning CT. The whole bladder was treated as CTV-H in the presence of multifocal disease. A 1-2 cm margin was added to CTV-H and GTV in all directions to account for organ motion and formed the planning target volume (PTV-H). IMRT was delivered via 4-10 MV photons using 5 to 7 static IMRT fields to PTV-H volume. The clinical target volume at intermediate risk (CTV-M) encompassed whole bladder in T2 disease and included pelvic nodal regions (including hypogastric, obturator, external iliac and perivesical nodes) in T3 and T4 disease. A 2-cm margin was given to CTV-M in the cranial and anterior directions and 1-cm in posterior, lateral and caudal directions, forming PTV-M. PTV-M was treated with 10 MV photons by 4-field 3D-CRT technique. All treatment planning was performed by Pinnacle version 9.0 (Philips Healthcare, Bothell, WA).

RT was initiated 4 to 8 weeks after urological procedure. Patients were treated with the same requirements as in the planning phase. A median total dose of 59.4Gy (range, 44.5 to 66.6Gy) was delivered to the PTV-H, and the PTV-M was irradiated with a median total dose of 40.0Gy (range, 36.0 to 54.0Gy) at 1.8-2.0 Gy per daily fraction. The prescribed doses should cover at least 95% of PTV volumes. The dose constraints were V_55_ < 50% for rectum, and D_max_ <45Gy to both bowels and femoral heads.

Concurrent platinum-based chemotherapy was offered and consisted of cisplatin (30 mg/m^2^/week) for patients without impaired renal function defined as glomerular filtration rate (GFR) >50mL/min, or carboplatin (100 mg/m^2^ on days 1, 15, 31) for patients with renal function impairment. The treatment-related characteristics were summarized in Table 1.

**Table 1:** **Patient, tumor and treatment-related characteristics**

Characteristics	Value
Age, years, median (range)	69 (49–92)
Gender	
Male	55 (78.6%)
Female	15 (21.4)
History of prior TUR-BT	
Initial treatment	50 (71.4%)
Recurrence after previous TURBT	20 (28.6%)
ECOG Performance status	
0	26 (37.1%)
1	43 (61.5%)
2	1 (1.4%)
Comorbidities	
No	20 (28.6%)
Yes	50 (71.4%)
Pathology	
Transitional cell carcinoma	65 (92.9%)
Adenocarcinoma	1 (1.4%)
Other histology or missing record	4 (5.7%)
Grade	
High	55 (78.6%)
Low	1 (1.4%)
Missing record	14 (20.0%)
Clinical T stage	
T2	41 (58.6%)
T3	16 (22.9%)
T4	13 (18.6%)
Focality	
Multifocal	26 (37.2%)
Single	44 (62.8%)
Hydronephrosis	
No	39 (55.7%)
Yes	31 (44.3%)
Procedure before radiotherapy (RT)	
Transurethral resection (TUR)	64 (91.4%)
Partial cystectomy	4 (5.7%)
Cystoscopic biopsy only	2 (2.9%)

### Follow-up and evaluation

Patients were observed at 3-month intervals for the first 3 years and every 6 months thereafter. Post-treatment follow-up consisted of pertinent medical history, physical examination, urine cytology, cystoscopy, and radiological evaluation as clinically indicated. Tumor response was evaluated using the Response Evaluation Criteria in Solid Tumors (RECIST) [15] both cystoscopically and radiographically (either CT or MRI) three months after treatment. Complete response (CR) was defined as the absence of detectable tumor as well as negative urine cytology. Among patients with less than CR, cystectomy was offered by the treating physicians. Salvage therapy for non-muscle invasive recurrences consisted of TUR-BT followed by intravesical therapy for non-muscle invasive recurrence and radical cystectomy for muscle-invasive recurrences. However, the final decision was determined at the discretion of urologist and patient’s own will. In case of distant failure, combination cisplatin and gemcitabine was offered as first line chemotherapy. Evaluation of late treatment-related toxicity was performed according to the toxicity criteria of the Radiation Therapy Oncology Group (RTOG) and the European Organization for Research and Treatment of Cancer (EORTC) [16, 17].

### Statistics

At the time of analysis, the median follow-up for the entire group was 24months. Survival was measured from the day of start of RT to the date of death or the most recent follow-up visit. The OS and PFS was calculated using the method of Kaplan-Meier and curve comparison by log-rank test. Univariate and multivariate analysis by Cox proportional hazards model were performed to determine clinicopathological factors with prognostic value for OS. A *p*-value <0.05 (two-sided) was considered significant in all of the statistical testing. The statistical analysis was performed using Statistical Package for Social Sciences software for Windows version 13.0 (SPSS, Inc, Chicago, IL).

## Results

### Tumor response after radiotherapy

Among the 70 patients, 64 patients were evaluable for response (3 patients died of non-cancer comorbidities shortly after treatment, and 3 patients refused cystoscopic evaluation). Of the evaluable 64 patients, 78.1% (50 patients) achieved CR (Table 2). Of the remaining patients with non-CR, two (3.1%) had partial response (PR), three (4.7%) had stable disease, and nine (11.4%) had progressive disease. Although radical cystectomy was offered to all patients with less than CR, none of the patients underwent this procedure due to patients' refusal or medical inoperability.

**Table 2:** **Tumor response after radiotherapy**

	Evaluable patients (n = 64)
Complete response (CR)	50 (78.1%)
Partial response (PR)	2 (3.1%)
Stable disease (SD)	3 (4.7%)
Progressive disease (PD)	9 (14.1%)

### Survival and bladder preservation

The median survival was 64.6 months after a median follow-up of 24 months. OS and PFS at 2 year were 65.7% and 51.9%. Local-regional control and distant metastasis free survival at 2 year were 69.8% and 73.5%, respectively. Bladder cancer specific survival and survival with intact bladder at 2 year were 77.3% and 64.2% respectively. The clinical outcomes at 2 year and 5 year were illustrated in Table 3.

**Table 3:** **Clinical outcomes**

	2-year rates	5-year rates
Overall survival	65.7%	50.8%
Bladder cancer specific survival	77.3%	67.3%
Survival with intact bladder^*^	64.2%	49.0%
Local-regional control	69.8%	61.7%
Distant-metastasis-free survival	73.5%	67.1%
Progression-free survival	51.9%	39.9%

*Only one patient with local failure received radical cystectomy as salvage treatment.

**Figure 1:**
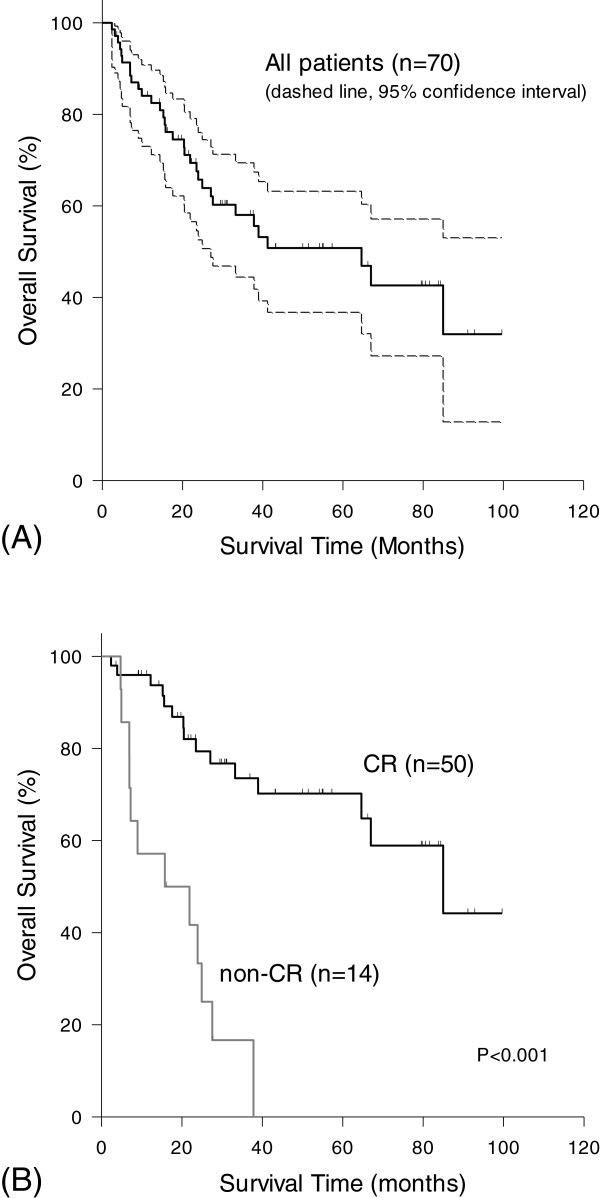
**Overall survival of all patients and comparison between CR and non-CR patients. (A)** The median survival, 2-year and 5-year OS rate of all patients were 64.6 months (95% CI, 32.3 to 97.0 months), 65.7% (95% CI, 52.6% to 76.0%), 50.8%. (95% CI, 36.7% to 63.2%). **(B)** Two-year overall survival rate was statistically higher among those with complete response than those without (79.4% [95% CI, 67.2% to 91.6%] vs. 33.3% [95% CI, 7.6% to 59.9%]; p < 0.001).

Univariate analysis of 12 treatment-related factors influencing OS was shown in Table 4. Age, performance status, T stage, prescribed dose to primary site and CR demonstrated significant hazard ratio on OS. Multivariate analysis showed age, performance status and CR as independent predictors on two-year overall survival (Table 4). Two-year overall survival rate was statistically higher among those with complete response than those without (79.4% [95% CI, 67.2% to 91.6%] vs. 33.3% [95% CI, 7.6% to 59.9%]; p < 0.001), while the median survival was 85.0 and 15.8 months (p < 0.001) (Figure 1).

**Table 4:** **Univariateand multivariate analysis for prognostic factors on overall survival**

Univariate analysis			
	**Hazard ratio**	**95% confidence interval**	**P value**
Male gender	0.730	0.327-1.627	0.442
Age, years	1.035	1.001-1.069	0.039
History of TURBT	0.986	0.465-2.087	0.971
ECOG performance status	3.182	1.388-7.291	0.006
Comorbidities	2.264	0.929-5.513	0.072
Clinical T stage	1.565	1.010-2.421	0.045
Hydronephrosis	1.825	0.901-3.692	0.095
RT duration, days	1.016	0.991-1.041	0.196
Concurrent chemotherapy	0.587	0.271-1.268	0.175
Prescribed doses to bladder tumor bed, Gy	0.914	0.858-0.972	0.005
Prescribed doses to whole bladder, Gy	1.001	0.999-1.001	0.245
Complete response (CR)	0.155	0.066-0.358	<0.001
**Multivariate analysis**			
Age	1.064	1.023-1.107	0.002
Performance status	2.840	1.133-7.119	0.026
Complete response (CR)	0.098	0.040-0.240	<0.001

### Failure pattern

**Figure 2:**
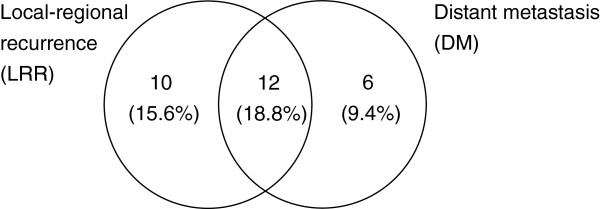
**Failure pattern.** The numbers in the circles were the patient numbers of relevant failure patterns. The percentage of relevant failure patterns were shown in the parentheses.

Of the 70 patients, isolated local-regional recurrence was observed in 10 patients (15.6%); isolated distant metastasis was observed in seven (9.4%); while both local-regional and distant metastasis developed in 12 patients (18.8%) (Figure 2). Of those 50 patients with CR, local recurrence occurred in 10 patients during the course of follow-up. Among whom six were muscle invasive, three were superficial, and the remaining one had missing histology record (Table 5). Among patients who developed muscle invasive recurrences, only one patient ultimately underwent radical cystectomy as salvage treatment, while the others refused salvage cystectomy or were medically inoperable due to old age or comorbidity. None of the patients with superficial recurrences died, whereas three of the invasive recurrences did. Distant metastasis developed in one of the surviving patients with muscle invasive recurrences.

**Table 5:** **Pattern of local recurrence in CR patients (n = 10)**

	Patient number	Status after salvage treatment
Superficial		
Papillary non-invasive carcinoma	1 (10%)	Alive with NED: 1 patient
Flat carcinoma in situ	2 (20%)	Alive with NED: 2 patients
Invasive urothelial carcinoma	6 (60%)	Alive with NED: 2 patients
		Alive with distant mets: 1 patient
		Expired: 3 patients
Missing record	1 (10%)	Expired: 1 patient

### Acute toxicity and chronic sequelae

All of the radiation induced acute genitourinary (GU) and gastrointestinal (GI) toxicities and were limited to grade 2 (Table 6), and manageable. Acute grade 2 GU toxicities accounted for 28.6% and grade 2 GI toxicities in 11.4%. Chronic GU and GI toxicities of grade 2 or more were encountered in 11 and 4 patients, respectively, while no patient with ≧ grade 3 GI toxicity. Chronic grade 3 GU toxicity developed in one patient and manifested as gross hematuria and needed repeated blood transfusion. No patient died from toxicities.

**Table 6:** **Toxicities**

	Grade 1	Grade 2	Grade 3	Grade 4	Grade 5
Acute GU toxicity	21 (30%)	20 (28. 6%)	0 (0%)	0 (0%)	0 (0%)
Acute GI toxicity	8 (11.4%)	8 (11.4%)	0 (0%)	0 (0%)	0 (0%)
Late GU toxicity	22 (31.4%)	10 (14.3%)	1 (1.43%)	0 (0%)	0 (0%)
Late GI toxicity	1 (1.43%)	4 (5.7%)	0 (0%)	0 (0%)	0 (0%)

## Discussion

A trimodality strategy for muscle-invasive bladder cancer as the main treatment strategy for muscle-invasive bladder cancer therapy has gained its popularity over time. However, an upfront CCRT approach without NAC is relatively infrequent.

Despite the relatively low dose density of chemotherapy in CCRT phase and a low radiation dose to the whole bladder in this study, our result of a 5 year OS of 50.8% compared reasonably to most prospective trials [7, 9, 18–20] and cystectomy series [21, 22]. A clinical CR rate of 78.1% and 5 year bladder intact survival of 49.0% in this study also compared reasonably to most upfront CCRT trials. Although NAC with platinum-based regimen before cystectomy compared to cystectomy was associated with a 5% survival benefit, the possible benefit of NAC before definitive CCRT has not been firmly established. Most NAC trials were single-armed [8, 10, 23, 24], with one randomized trial demonstrating no statistically significant benefit [4].

The presence of CR was important in predicting OS was not an exception in ours as most prospective [4, 19, 25]and retrospective series [5, 26, 27]. In one retrospective series, CR in stage IV bladder cancer had significantly better prognosis than those with stage II or III disease but with no CR [26]. In this study, those who failed to achieve CR fared poorly without radical cystectomy. None of the non-CR patients was alive at 5 year. The normogram developed at Massechuses General Hospital (MGH) can be used to predict response to CCRT, in which hydronephrosis was one of the poor response indicators [28]. However, the presence of hydronephrosis might not be a contraindication to bladder organ preservation treatment, because a significant proportion of patients (43.3%) in our study presented with hydronephrosis and the rate of CR was compatible to most publications. Biomarkers might be useful to better select patients for a trimodality approach and should be evaluated in between prospective trials [29].

A total of 10 local recurrences (20%) developed among 50 CR patients. Thirty percent of them were superficial recurrence whereas most of them were still muscle-invasive. The proportion of muscle invasive recurrences in our study seemed higher than other reports. In RTOG 95-06, the number of muscle invasive recurrence were only half of superficial recurrences [19]. Our results of a 2-year bladder intact survival rate of 64.2% compared favorably to the result of RTOG 8903 that had 99 patients without hydronephrosis [4]. In our study, the overall 20% recurrence rate in CR patients is close to that observed in the Paris group, which reported a bladder recurrence rate of 17% [30].

The optimal dose and fractionation of radiation may be important in achieving a lasting response [31]. Hyperfractionation and hypofractionation were evaluated in prospective trials [19, 32]. Hypofractionated approach had better gone through whole course IMRT in order to spare normal organ at risk as much as possible. Current radiation protocol for bladder preservation regarded whole bladder dose of 54Gy and tumor bed 64-66Gy to be safe [33]. As the results of BC2001 showed no statistically increased toxicity with whole bladder irradiation to reduced high dose volume irradiation [34]. Organ motion have attributed to the uncertainties in target delineation and radiation delivery for bladder cancer treatment. Our strictly enforced bladder filling policy protocol without image-guided technique, although quite successful, should be replaced by imaged guided technique, especially for the part of tumor bed boost. The value of image guided radiotherapy (IGRT) of bladder cancer should be the mainstay of future work [35–37].

Unlike to the routine pelvic irradiation among RTOG protocols [3, 4, 9, 19], our bladder-only approach for T2 tumors and pelvic nodal coverage for T3, T4 lesions seemed appropriate. In a large retrospective series by Stein et al [38], incidence of lymph node involvement was closely related to the T staging of the primary bladder tumor; the incidences of lymph node involvement were 18% and 42% for T2 and T4 tumors, respectively. Our radiation protocol resulted in low locoregional failure rate (15.6%). In additional to the low incidence of regional failure, our study also demonstrated very low incidence of late grade toxicity (1.43% of late GU toxicity and 0% of late GI toxicity). Acute grade 3 or more toxicity was not seen. The bladder-only irradiation in combination with 3D-CRT and IMRT to T2 disease may be a sound alternative to pelvic irradiation.

The distant metastasis free survival rate in our study is 73.5% and 67.1% at 2- and 5-year, respectively. Extravesicle diseases were consistently associated with high rates of distant metastasis. The value of adjuvant chemotherapy in bladder preservation was investigated in RTOG 97-06 [32]. Although the result was promising, less than half of the enrolled patients completed the designated treatment. Adjuvant chemotherapy was not part of our treatment strategy in patients without pelvic node metastasis, even though a substantial proportion of extravesicle tumors in our study (T3 + T4 = 41.5%). Yet this did not jeopardize our OS rate. Unless randomized trial has proven NAC followed by CCRT and followed by chemotherapy is better CCRT alone, our upfront CCRT strategy will be continued.

## Conclusions

Aim of bladder preservation therapy is to offer a quality of life treatment and avoid potential morbidity from chemotherapy or radiotherapy without compromising bladder preservation rate. Upfront CCRT with mild weekly cisplatin regimen without NAC or adjuvant chemotherapy could provide reasonable CR rate, OS and bladder preservation rate in T2-4N0 stage bladder cancer patients.

## Abbreviations

TURBT: Transurethral resection of bladder tumor
RT: Radiotherapy
CCRT: Combined chemotherapy and radiotherapy
3D-CRT: 3D Conformal radiotherapy
IMRT: Intensity modulated radiotherapy
NAC: Neoadjuvant chemotherapy
RTOG: Radiation Therapy Oncology Group
OS: Overall survival
PFS: Progression-free survival
CR: Complete response
GU: Genitourinary
CT: Computed tomography
MRI: Magnetic resonance imaging
AJCC: American Joint Committee on Cancer
GTV: Gross tumor volume
CTV-H: Clinical target volumes at high risk
PTV-H: Planning target volume at high risk
CTV-M: Clinical target volume at intermediate risk
PTV-M: Planning target volume at intermediate risk
GFR: Glomerular filtration rate
RECIST: Response Evaluation Criteria in Solid Tumors
EORTC: European Organization for Research and Treatment of Cancer
SPSS: Statistical Package for Social Sciences software
PR: Partial response
GI: Gastrointestinal
MGH: Massechuses General Hospital
IGRT: Image guided radiotherapy.
